# Macrophage Trogocytosis of Tumor Cells Drives the Immunosuppression of Tumor Microenvironment

**DOI:** 10.1002/advs.77518

**Published:** 2026-09-30

**Authors:** Chao Liang, Jiaqi He, Wan‐Ru Zhuang, Yao Lei, Yanru Jiang, Xianbin Ma, Pan Liu, Zhujun Jiang, Yongheng Li, Hai‐Yan Xie

**Affiliations:** ^1^ State Key Laboratory of Natural and Biomimetic Drugs School of Pharmaceutical Sciences, Chemical Biology Center Peking University Beijing China; ^2^ Department of Pancreatic Cancer Tianjin Medical University Cancer Institute and Hospital National Clinical Research Center for Cancer Tianjin Key Laboratory of Digestive Cancer Key Laboratory of Cancer Prevention and Therapy Tianjin China; ^3^ Division of Life Science and Medicine University of Science and Technology of China Hefei China; ^4^ School of Medical Technology Beijing Institute of Technology Beijing China; ^5^ Key laboratory of Carcinogenesis and Translational Research (Ministry of Education/Beijing) Department of Radiation Oncology Peking University Cancer Hospital and Institute Beijing China

**Keywords:** cortical stiffness, immunosuppressive heterogeneity, T cell exhaustion, trogocytosis, tumor‐associated macrophages

## Abstract

Despite considerable advances in cancer immunotherapy, the persistent immunosuppressive tumor microenvironment (TME) seriously limits its performance in solid tumors. Here, we reveal that trogocytosis—an intercellular membrane transfer process—between tumor‐associated macrophages (TAMs) and cancer cells serves as a critical mechanism driving this immunosuppression. We demonstrate that trogocytosis efficiency, inversely correlated with tumor cell cortical stiffness, varies substantially across cancer types and dictates immunosuppressive intensity through upregulation of CXCL5 and Arg‐1, leading to CD8^+^ T cell exhaustion. Importantly, this mechano‐immunological axis is conserved in humans, as evidenced by study with human cell lines and a strong correlation between trogocytosis markers and T cell exhaustion in clinical patient samples. Increasing tumor cell stiffness with simvastatin effectively suppressed trogocytosis, reversed T cell dysfunction, and synergistically enhanced the efficacy of adoptive T cell therapy. These findings establish macrophage trogocytosis as a fundamental driver of immunosuppressive heterogeneity and highlight its targeting as a promising strategy to potentiate cancer immunotherapy.

## Graphical abstract

Trogocytosis efficiency varies significantly among different tumor cells and is inversely related to their cortical stiffness; this efficiency dictates the extent of immunosuppression via upregulation of CXCL5 and Arg‐1, ultimately driving CD8^+^T cell exhaustion and tumor escape.

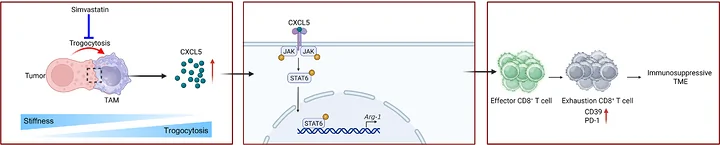

## 1 Introduction

Although immunotherapy has extended overall survival for a range of malignancies, durable responses remain limited to a minority of patients due to a number of heterogeneous factors [1, 2]. Especially, the immunosuppressive tumor microenvironment (TME) is of central importance for malignant progression, metastatic dissemination, and immune evasion, seriously impairing immunotherapy outcome [3, 4, 5, 6]. TME is orchestrated by diverse dynamic interplays, with intercellular cross‐talking standing out as the critical regulatory mechanism for its establishment and perpetuation [7, 8, 9]. Although various cell‐cell interaction pairs and paradigms have been revealed, myriad cellular interactions are still unknown, which seriously impedes cancer immunotherapy development [10, 11].

Recently, trogocytosis—a smart process that one cell “nibbling on” another to transfer membrane and associated cytosol—has emerged as an important model of intercellular communication and played a crucial role in immune regulation within TME [12, 13, 14]. Through trogocytosis, NK cells acquire antigen presentation capabilities [15, 16], and T cells acquire immune‐suppressive molecules [17, 18]. Trogocytosis also affects therapeutic strategies, particularly CAR NK/T cell therapy, by causing antigen loss from target cancer cells and impacting cell functionality [19, 20, 21]. Tumor‐associated macrophages (TAMs), primarily of the M2‐like phenotype, are one of the most abundant immune cells in TME and play a pivotal role in TME remodeling [22, 23, 24]. They typically do not engage in phagocytosis and digestion of living tumor cells; however, they actively interact with different cells in TME, promoting tumor growth, invasion, and metastasis [25, 26, 27]. Although macrophages are known to mediate trogocytosis of antibody‐opsonized tumor cells in vitro, it is unclear whether they use this mechanism to interact with native tumor cells in the TME [28, 29, 30]. Moreover, all current studies have focused on discerning different forms of trogocytosis. Nevertheless, the heterogeneity of this process across tumor types remains unclear.

Here, we report that TAM engage in trogocytosis with cancer cells in both mouse tumor models and human clinical tumors. Moreover, the extent of this interaction varies substantially across tumor types, decreasing sequentially in 4T1 breast cancer, B16F10 melanoma, and MC38 colorectal cancer. This heterogeneity is regulated by the cortical stiffness of tumor cells, where lower cell stiffness leading to more trogocytosis. This process, in turn, induces heterogeneous expression of chemokines (e.g., CXCL5, IL‐33, CCL2, IL‐10) and metabolic enzymes (e.g., Arg‐1, Spk1, Net5) in TAM, promoting monocyte recruitment and intensifying immunosuppression within the TME. Accordingly, increased trogocytosis correlates with reduced CD8^+^ T cell infiltration and more severe T cell exhaustion, and trogocytosis inhibition potently boosted the antitumor response of both endogenous and exogenous T cells. Thus, TAM trogocytosis significantly contributes to immunosuppressive heterogeneity in the TME, presenting a new therapeutic avenue for immunotherapy.

## 2 Results

### 2.1 Tumor‐Type‐Specific Hierarchy of Trogocytosis by Macrophages

Despite the well‐established role of macrophages as phagocytic cells, the phagocytic capacity of M2‐like Raw264.7 macrophages for living tumor cells was negligible during a 6‐h co‐incubation period (Figure S1A), which is consistent with previous reports [31, 32]. To investigate whether M2‐like Raw264.7 macrophages engage in trogocytosis with tumor cells, we biotinylated membrane proteins of 4T1 tumor cells using an NHS‐based conjugation (Figure S1B). The labeled cells were then co‐incubated with M2‐like Raw264.7 macrophages (Figure 1A). Flow cytometry detected substantial membrane transfer from tumor cells to M2 macrophages, and 73.8% of M2‐like Raw264.7 macrophages becoming biotin‐positive after 6 h. This transfer was virtually abolished when M2‐like Raw264.7 macrophages and tumor cells were separated by a Transwell system, confirming that direct cell contact was essential and also ruling out significant contribution from exosomes. Moreover, treatment with latrunculin A—an actin polymerization inhibitor that blocks trogocytosis—reduced biotin transfer by 5.5‐fold (Figure 1B,C). These results revealed efficient trogocytosis between M2‐like Raw264.7 macrophages and 4T1 tumor cells without opsonization. Inspired by the occurrence of trogocytosis across cell types, we then explored the trogocytosis between M2‐like Raw264.7 macrophages and other tumor lines, including B16F10 melanoma and MC38 colorectal carcinoma. Interestingly, marked differences were observed: the percentage of biotin‐positive M2‐like Raw264.7 macrophages was 42.2% for B16F10 and 16.3% for MC38, compared to 73.8% for 4T1. Consistent with this trend, both CLSM imaging and flow cytometry imaging showed a steadily decreased biotin signal on M2‐like Raw264.7 macrophages in the order of 4T1> B16F10> MC38 (Figure 1D,E and Figure S1C). This variance was not due to differences in tumor cell number or viability after the 6‐hour co‐culture (Figure S1D). The next real‐time live‐cell confocal imaging results showed that discrete biotin foci were rapidly transferred from 4T1 tumor cells to M2‐like Raw264.7 macrophages within 30 min and accumulated in the following 150 min. In contrast, transfer from B16F10 and MC38 cells began only at about 180 and 360 min, respectively (Figure 1F,G). Quantification results revealed that the proportion of cell contacts resulting in trogocytosis was 74% for 4T1, 48% for B16F10, and 21% for MC38 within 360 min (Figure 1H).

**FIGURE 1:**
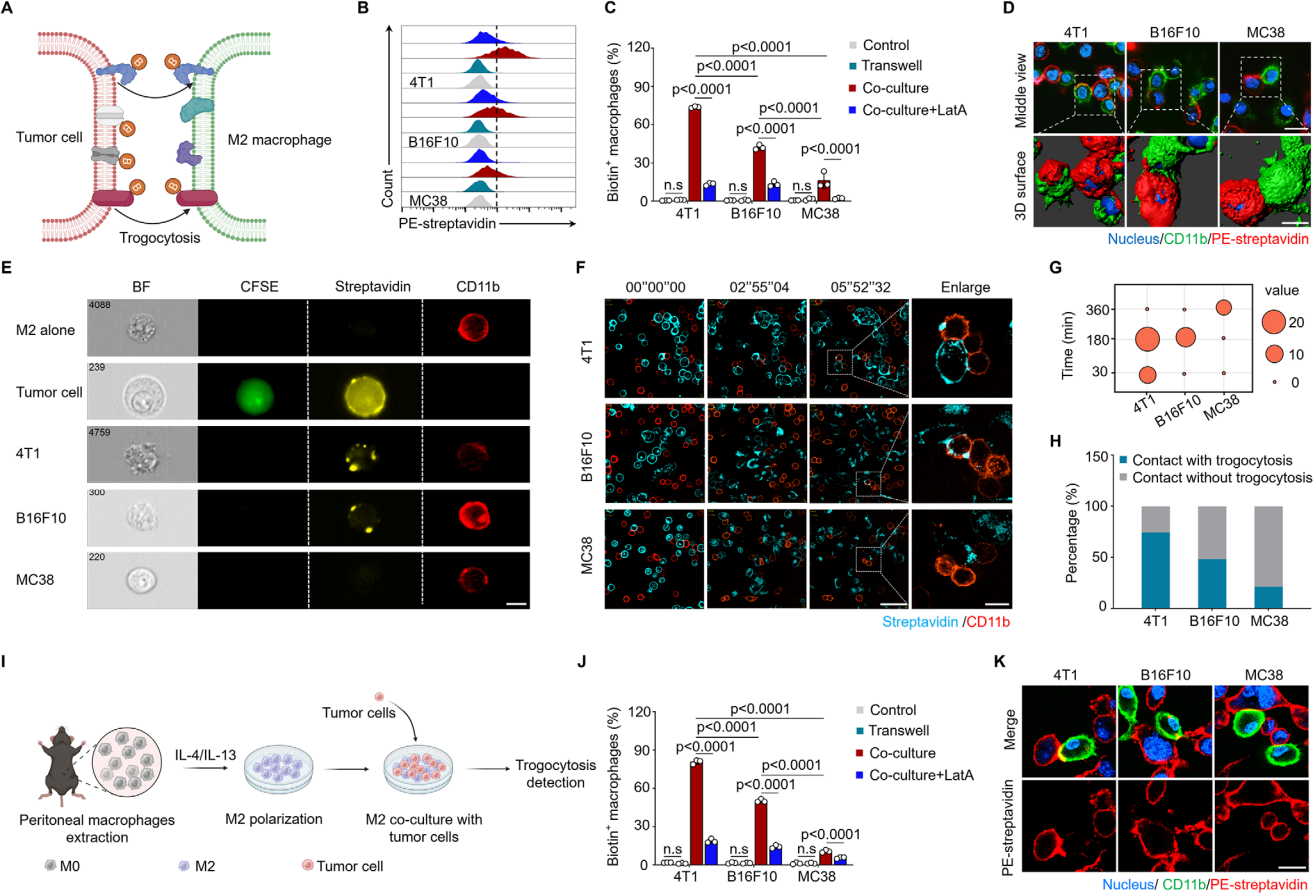
Tumor‐type‐specific hierarchy of trogocytosis by macrophages. (A) Schematic illustration of trogocytosis between M2 macrophages and tumor cells. (B,C) Representative flow cytometry and corresponding quantification of biotin‐positive M2‐like Raw264.7 macrophages after 6 h co‐culture. Cells were stained with streptavidin to detect acquired biotinylated proteins (*n* = 3). (D) Representative CLSM and three‐dimensional reconstitution images of trogocytosis between M2‐like Raw264.7 macrophages and three kinds of tumor cells. Nucleus (Blue), M2 macrophages (Green), tumor cells (Red). Scale bar: 20 µm (CLSM images) or 10 µm (three‐dimensional reconstitution images). (E) Representative flow cytometry imaging of M2‐like Raw264.7 macrophages co‐cultured with three tumor cells, stained with anti‐CD11b and streptavidin. Scale bar: 10 µm. (F) Time frames of confocal live imaging. Cyan foci represent proteins acquired from tumor cells via trogocytosis, with dashed squares denoting the magnified areas. Scale bar: 40 µm (original images) or 10 µm (enlarged images). (G,H) Quantification of trogocytosis frequency from live imaging in (F). (I) Schematic diagram of the experimental design for investigating trogocytosis between primary M2 peritoneal macrophages and tumor cells. (J) Representative flow cytometry and corresponding percentage of biotin‐positive primary M2 peritoneal macrophages after co‐culture for 6 h. M2 macrophages were stained with PE‐streptavidin (*n* = 3). (K) Representative CLSM of trogocytosis between primary M2 peritoneal macrophages and three kinds of tumor cells. Nucleus (Blue), M2 peritoneal macrophages (Green), tumor cells (Red). Scale bar: 10 µm. Data are presented as mean ± SD from biological replicates. Statistical significance was determined by one‐way ANOVA followed by Tukey's multiple comparisons test. n.s, no significance.

To validate these findings in a more physiologically relevant context, primary M2 macrophages derived from primary peritoneal macrophages and primary TAMs (F4/80^+^MHC‐II^−^) were also used (Figure 1I and Figure S1E). Both exhibited a tumor‐type‐dependent pattern of biotin acquisition from tumor cells (Figure 1J,K and Figure S1F). Taken together, these data demonstrated that M2 macrophages could engage in opsonin‐independent trogocytosis with tumor cells, and that the speed and efficiency of this process vary significantly across different tumor cells.

### 2.2 Membrane Stiffness Hierarchy Determines Trogocytosis Intensity

Trogocytosis facilitates the transfer of membrane fragments and associated components (e.g., surface proteins) from donor cells to receptor cells [33, 34, 35, 36]. Western blotting confirmed that cell contact was required for intercellular protein transfer, as it was observed after co‐incubation but not when tumor cells and M2‐like Raw264.7 macrophages were physically separated by a Transwell system. Consistent with the trogocytosis efficiency, protein transfer was most with 4T1 cells, markedly less in B16F10 cells, and minimal in MC38 cells (Figure 2A). For quantitative validation, we performed global label‐free proteomics (Figure 2B). A total of 61 tumor‐derived membrane proteins were identified in the 4T1 group, compared to 33 in the B16F10 group, and only 16 in the MC38 group (Figure 2C). The total protein intensity (TPI) in the 4T1 group was 2.8‐fold and 10.5‐fold higher than that in the B16F10 and MC38 groups, respectively (Figure 2D). Among the five proteins commonly transferred across all three tumor types, the level of desmoglein‐1 (Dsg‐1), a desmosomal adhesion molecule, correlated positively with trogocytosis efficiency (Figure 2E). This correlation was further supported by flow cytometry (Figure S2A), although Dsg‐1 expression was not the most on 4T1 surface, and no significant difference was observed at the mRNA level in M2‐like Raw264.7 macrophages after treatment with different tumor cells (Figure S2B,C).

**FIGURE 2:**
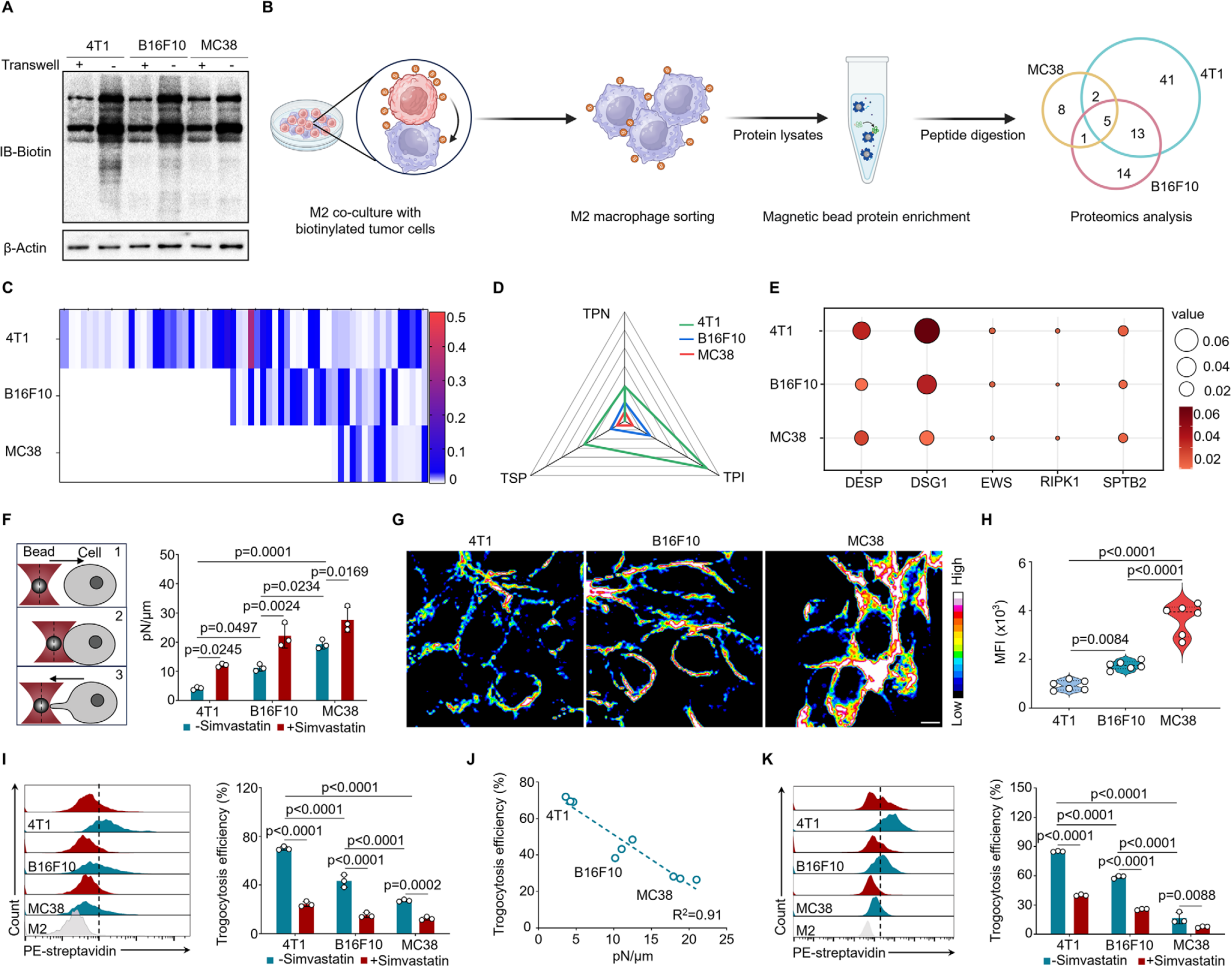
Membrane stiffness hierarchy determines trogocytosis intensity. (A) Western blot analysis of biotinylated protein acquisition in M2‐like Raw264.7 macrophages under varying co‐culture conditions. (B) Workflow for proteomics analysis of M2‐like Raw264.7 macrophages following co‐culture with tumor cells. (C) Quantification of surface protein acquisition by M2‐like Raw264.7 macrophages based on proteomic analysis. (D) Radar plot of fold‐change distributions across three dimensions: TPN (total membrane protein number), TSP (tumor specific proteins), TPI (total membrane protein intensity). (E) Comparative analysis revealing five shared proteins across tumor cell lines. (F) Schematic illustration of measuring tumor cells membrane stiffness via optical tweezer. 1. Approaching the cell with a microbead. 2. Attaching the microbead to the cell membrane. 3. Pulling the microbead to induce membrane deformation (left) and corresponding quantitative results of different tumor cell lines (right) (*n* = 3). (G) Representative F‐actin distribution in tumor cells stained with phalloidin‐PE. Scale bar: 10 µm. (H) Quantification of mean fluorescence intensity in tumor cells (*n* = 6). (I) Representative flow cytometry and corresponding quantification of biotin‐positive M2‐like Raw264.7 macrophages after 6 h of co‐culture. Cells were stained with streptavidin before analysis (*n* = 3). (J) Correlation between trogocytosis efficiency and membrane stiffness. (K) Representative flow cytometry and corresponding quantification of trogocytosis efficiency of primary peritoneal M2 macrophages following co‐culture with tumor cells with or without simvastatin treatment (*n* = 3). Data are presented as mean ± SD from biological replicates. Statistical significance was determined by one‐way ANOVA followed by Tukey's multiple comparisons test.

It is known that trogocytosis often involves specific ligand‐receptor interactions, and sialic acid‐Siglec binding is the glyco‐immune checkpoint between macrophages and tumor cells [37, 38]. However, disruption this binding through enzymatic desialylation did not significantly reduce trogocytosis, suggesting that this interaction is not essential in this process (Figure S2D,E). Recent reports suggest that tumor cell membrane stiffness can govern trogocytosis [29]. We therefore measured the cortical elasticity of different tumor cells using optical tweezers and observed a strong negative correlation between tumor cell stiffness and trogocytosis efficiency (R^2^ = 0.91, r = −0.95, p<0.0001). Specifically, MC38 cells exhibited the highest stiffness (19.26 pN/µm), being 1.7‐fold and 4.6‐fold stiffer than B16F10 (11.24 pN/µm) and 4T1 cells (4.17 pN/µm), respectively (Figure 2F). Since actin plays an important role in regulating membrane rigidity, we also quantified F‐actin density. Phalloidin‐PE staining showed that MC38 cells exhibited 4‐fold and 2.1‐fold higher F‐actin intensity than 4T1 cells and B16F10 cells, respectively, further support the inverse relationship between membrane stiffness and trogocytosis efficiency (Figure 2G,H). Given that membrane cholesterol content negative correlates with cortical stiffness [17, 39], we treated tumor cells with simvastatin, a clinically used cholesterol‐depletion agent, and found significant increased cortical stiffness across all tumor cells (Figure 2F). In consistent, trogocytosis efficiency was significantly reduced by 2.9‐fold in 4T1 cells, 2.8‐fold in B16F10 cells, and 2.2‐fold in MC38 cells (Figure 2I). This response maintained a strong negative correlation between cortical stiffness and trogocytosis (R^2^ = 0.83, r = −0.91, *p* = 0.0006) (Figure S2F). Similar reductions were observed when M2 macrophages derived from primary peritoneal macrophages replaced M2‐like Raw264.7 macrophages, with decreases exceeding 50% in 4T1 and B16F10, and 45.5% in MC38 groups (Figure 2K). Collectively, these results illustrated that tumor cell cortical stiffness is a key determinant of M2‐mediated trogocytosis and that pharmacological stiffening of tumor cells provides a viable strategy to suppress this process.

### 2.3 Trogocytosis‐Driven Macrophage Polarization and Recruitment Promotes T Cell Exhaustion

Trogocytosis typically triggers a cascade of signal activation and gene expression, which lead to metabolism alterations and cytokine release [40, 41, 42]. To investigate its impacts on M2 macrophages, we performed transcriptomic profiling of M2‐like Raw264.7 macrophages after 24‐h co‐culture with 4T1 tumor cells. Comparative analysis identified 1082 significantly upregulated genes (fold change >2; adjusted *p*<0.05) in the direct co‐culture system compared to the Transwell system (Figure 3A). Kyoto Encyclopedia of Genes and Genomes (KEGG) pathway analysis revealed that these differentially expressed genes (DEGs) were mainly enriched in metabolic pathways, cancer‐related pathways, PI3K‐Akt signaling, and cytokine‐cytokine receptor interaction (Figure 3B). Gene set enrichment analysis (GSEA) further verified these findings (Figure 3C,D). To explore transcriptional reprogramming associated with trogocytosis efficiency, we then evaluated the expression of key metabolic and cytokine signaling components selected from the DEGs significantly upregulated. Quantitative real‐time polymerase chain reaction (qRT‐PCR) analysis revealed that most selected genes were significantly upregulated across three tumor cell groups. Among them, the expression levels of *CXCL5* and *Arg‐1* showed strong positive correlations with trogocytosis efficiency (Figure 3E,F and Figure S3A, B. R^2^ = 0.89, r = 0.94, p = 0.0001 and R^2^ = 0.93, r = 0.96, p<0.0001, respectively;). The dependency of CXCL5 on trogocytosis efficiency was further verified by western blotting (Figure 3G). At the mRNA level, *CXCL5* expression increased by 504‐fold in 4T1 group but this upregulation was reduced by 49% following simvastatin pretreatment. In B16F10 group, the 143‐fold increase was suppressed by 68%, while in MC38 cells, the original 8‐fold upregulation reduced to only 3‐fold after treatment (Figure 3H). Similarly, *Arg‐1* expression increased by 24.5‐, 7.9‐, and 1.3‐fold in the 4T1, B16F10, and MC38 groups, respectively, and declined to 12.9‐fold, 1.6‐, and 0.9‐fold after simvastatin treatment (Figure 3I). Flow cytometry analysis yielded consistent results (Figure S3C). CXCL5 is a typical chemokine that can recruit monocytes through recognition with CXCR2 [43, 44, 45]. Consequently, the supernatant of M2‐like Raw264.7 macrophages and 4T1 tumor cells after incubation markedly promoted monocyte recruitment, which was clearly diminished in the B16F10, MC38, and simvastatin‐treated 4T1 groups (Figure 3J,K and Figure S3D). Moreover, the number of migrating monocytes showed a strong positive correlation with CXCL5 expression (Figure S3E, R^2^ = 0.90, r = 0.95, p = 0.0001).

**FIGURE 3:**
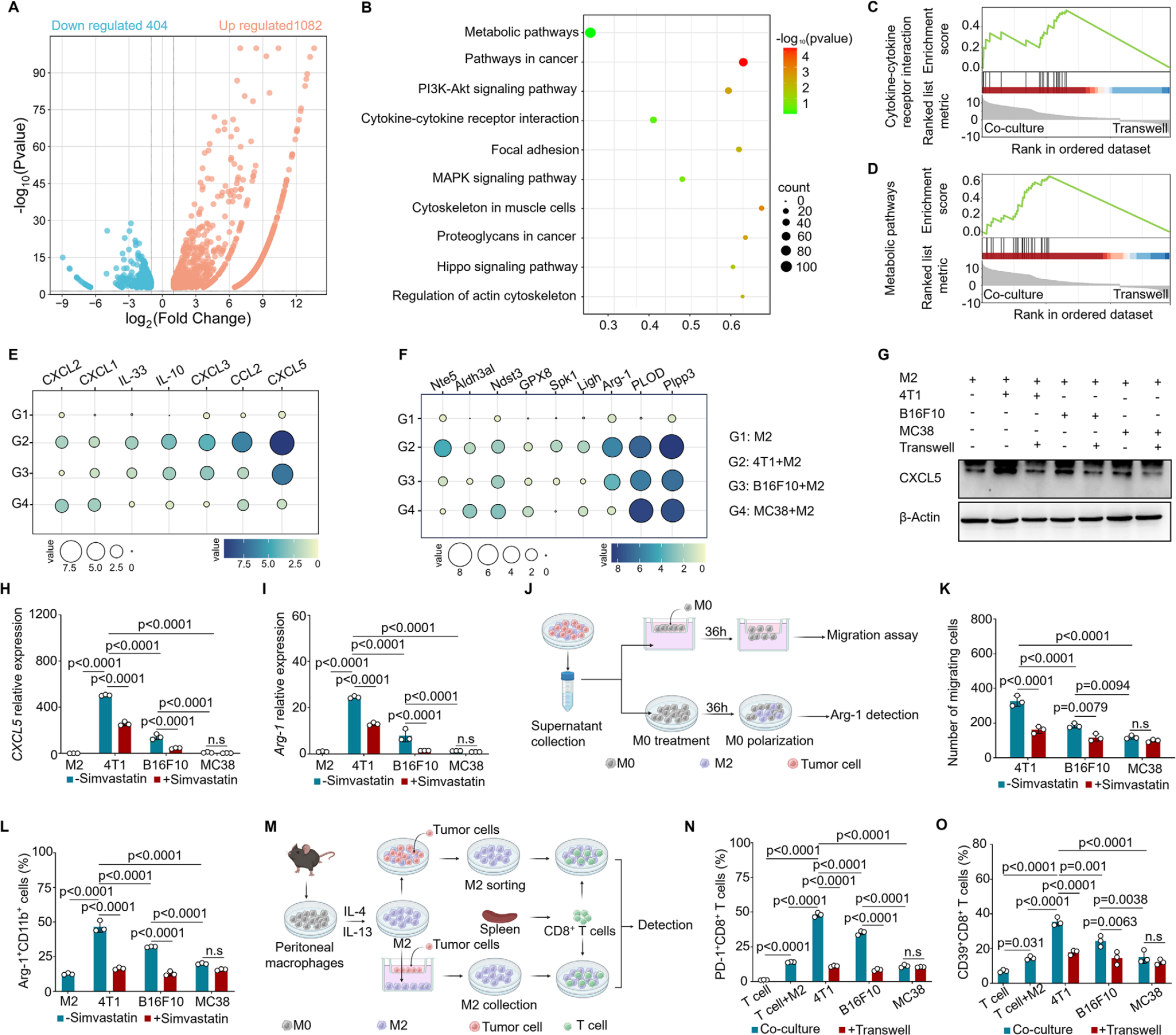
Trogocytosis‐driven macrophage polarization and recruitment promotes T cell exhaustion. (A) Volcano plot of gene expression changes in M2‐like Raw264.7 macrophages co‐cultured with 4T1 tumor cells versus untreated control. (B) KEGG pathway enrichment analysis of differentially expressed genes. (C,D) Gene set enrichment analysis (GSEA) showing upregulated cytokine‐cytokine receptor interactions (C) and metabolic pathways(D) in M2‐like Raw264.7 macrophages co‐cultured with 4T1 tumor cells. (E,F) Relative mRNA levels of cytokines (E) and metabolic regulators (F) in M2‐like Raw264.7 macrophages co‐cultured with different tumor cell lines. (G) Western blot analysis of CXCL5 expression in M2‐like Raw264.7 macrophages co‐cultured with different tumor cells. (H) Representative data showing *CXCL5* expression in M2‐like Raw264.7 macrophages after co‐culture with untreated or treated tumor cells (*n* = 3). (I) Representative data showing *Arg‐1* expression in M2‐like Raw264.7 macrophages after co‐culture with untreated or treated tumor cells (*n* = 3). (J,K) Experimental schematic (J) and Raw264.7 macrophage recruitment quantification under different supernatant treatments (K). (L) Quantification of Arg‐1 expression in Raw264.7 macrophages with different supernatant treatments (*n* = 3). (M) Scheme of T‐cell functional assays after primary M2 peritoneal macrophages co‐culture under different conditions. (N,O) Representative flow cytometry and corresponding quantification of PD‐1^+^CD8^+^T cells (P) and CD39^+^CD8^+^T cells (Q) (*n* = 3). Data are presented as mean ± SD from biological replicates. Statistical significance was determined by one‐way ANOVA followed by Tukey's multiple comparisons test. n.s, no significance.

It is reported that CXCL5 promotes macrophage polarization via activating the STAT3/STAT6 signal pathway, thereby inducing Arg‐1 expression [46, 47]. To test this, we treated M0 macrophages with supernatant from each group. Interestingly, the proportion of Arg‐1^+^ macrophages increased from 12.6% in the M2‐ only control group to 46.7% with 4T1 supernatant, and only to 16.6% with simvastatin‐treated 4T1‐supernatant. B16F10 supernatant increased Arg‐1^+^ macrophages to 32.2%, whereas MC38 supernatant showed negligible effects (Figure 3L). Given that Arg‐1 upregulation is indicative of immunosuppressive activity, we then evaluated the functional impact of trogocytosis‐educated M2 macrophages on T cells. Primary M2 peritoneal macrophages were co‐cultured with tumor cells, sorted, and then incubated with activated splenic CD8^+^ T cells. T cells co‐cultured with 4T1‐educated M2 showed significant upregulation of exhaustion markers PD‐1 (42.6‐fold) and CD39 (5.0‐fold) relative to pristine T cells (Figure 3M–O). These increases were progressively lower in the B16F10 and MC38 groups. The percentage of PD‐1^+^CD8^+^ and CD39^+^CD8^+^ T cell showed strong positive correlations with trogocytosis efficiency (R^2^ = 0.93, r = 0.93, p<0.0001 and R^2^ = 0.76, r = 0.94, p = 0.0002, respectively; Figure S3F,G). When M2 macrophages were cultured with tumor cells in Transwell systems—preventing trogocytosis—the upregulation of PD‐1 and CD39 was also hindered. In summary, trogocytosis heterogeneity in M2 macrophages toward different tumor cells leads to distinct gene expression profiles, which in turn drive differences in monocyte recruitment and polarization capacity, ultimately shaping the immunosuppressive landscape of the TME.

### 2.4 Simvastatin Disrupts Trogocytosis and Reverses T‐Cell Exhaustion in Vivo

After recognizing the trogocytosis heterogeneity and its effect in vitro, we sought to investigate its functional impact in vivo. We established subcutaneous tumor models of breast cancer (4T1), melanoma (B16F10), and colorectal cancer (MC38), and monitored the transfer of Dsg1—a protein to TAMs. Immunofluorescence staining revealed substantial Dsg1 transfer from tumor cells to TAMs in breast cancer, with progressively less transfer in melanoma and colorectal cancer (Figure 4A). Flow cytometry quantification showed that Dsg1^+^ TAMs accounted for 35.2% of TAMs in breast cancer, 18.2% in melanoma, and only 1.6% in colorectal cancer. Simvastatin treatment significantly reduced these percentages across all models, confirming its inhibitory effect on trogocytosis in vivo (Figure 4B,C and Figure S4A). On the other hand, Both *CXCL5* and *Arg‐1* expression levels strongly correlated with trogocytosis efficiency (Figure S4B, C, R^2^ = 0.88, r = 0.94, p<0.0001 and R^2^ = 0.81, r = 0.90, p<0.0001, respectively), and simvastatin suppressed their upregulation, particularly in breast cancer and melanoma (Figure 4D).

**FIGURE 4:**
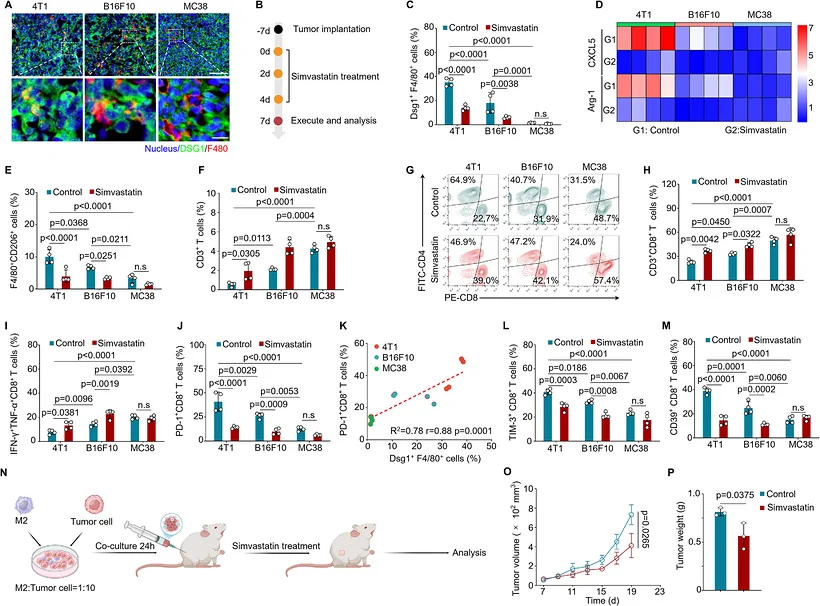
Simvastatin disrupts trogocytosis and reverses T‐cell exhaustion in vivo. (A) Representative immunofluorescence images of trogocytosis in different tumor tissues. Nucleus (Blue), Dsg1 (Green), and F4/80 (Red). Scale bar: 100 µm (original images) or 10 µm (enlarged images). (B) Scheme of simvastatin treatment. (C) Flow cytometric analysis of Dsg1^+^ TAMs in tumors with or without simvastatin treatment (*n* = 4). (D) Heatmap of *CXCL5* and *Arg‐1* expression in tumors from simvastatin‐treated versus untreated groups. (E) Percentage of F4/80^+^CD206^+^ cells in tumors with or without simvastatin treatment (*n* = 4). (F) Flow cytometric analysis of CD3^+^ T cells infiltration in tumors with or without simvastatin treatment (*n* = 4). (G,H) Representative flow cytometry and corresponding quantification of CD3^+^CD8^+^ T cells in different tumors with or without simvastatin treatment (*n* = 4). (I) Flow cytometric analysis of TNFα^+^IFNγ^+^ CD8^+^T cells in tumors with or without simvastatin treatment (*n* = 4). (J–M) Flow cytometric analysis of PD‐1^+^CD8^+^ T cells (J), correlation between Dsg1^+^F4/80^+^ cells and the percentage of PD‐1^+^CD8^+^ T cells (K), quantification of TIM‐3^+^CD8^+^ T cells (L) and CD39^+^CD8^+^ T cells (M) in different tumor tissues with or without simvastatin treatment (*n* = 4). (N) Experimental timeline. (O,P) Growth curves (O); Tumor weights (P) (*n* = 3). Data are presented as mean ± SD from biological replicates. Statistical significance was determined by one‐way ANOVA followed by Tukey's multiple comparisons test. n.s, no significance. Unpaired two‐tailed Student's t test for (O, P).

The proportion of CD206^+^ M2 macrophages was also positively correlated with trogocytosis (R^2^ = 0.81, r = 0.94, p<0.0001; Figure 4E and Figure S4D), while CD3^+^ T cell infiltration exhibited a negative correlation (R^2^ = 0.87, r = −0.93, p<0.0001; Figure 4F and Figure S4E). Simvastatin treatment increased CD3^+^ T cell infiltration in all three models (Figure 4F), suggesting that trogocytosis inhibition helps restore T cell presence in the TME. Furthermore, simvastatin markedly enhanced CD3^+^CD8^+^ T cell infiltration and effector function while alleviated its exhaustion. CD3^+^CD8^+^ T cell abundance increased by 1.6‐fold in breast cancer (Figure 4G,H), the fraction of TNF‐α^+^IFN‐γ^+^CD8^+^ T cells‐ a key indicator of cytotoxic activity‐ significantly rose following treatment (Figure 4I and Figure S4F). In contrast, the percentage of PD‐1^+^CD8^+^ T cells, which correlated strongly with trogocytosis efficiency (R^2^ = 0.78, r = 0.88, p = 0.0001), was significantly reduced in all three models (Figure 4J,K). Similar reductions were also observed for other T cell exhaustion markers, TIM‐3 and CD39, across all models (Figure 4L,M). Alleviated T cell exhaustion would improve the tumor inhibition capability of endogenous T cells. For confirmation, 4T1 cells and M2 macrophages were co‐cultured and then subcutaneously co‐injected into mice. Simvastatin was administered every 2 days when tumors reached 50 mm^3^ (Figure 4N). Mice were euthanized on day 19 post‐implantation for evaluation. Compared with untreated mice (Control group), tumor volume and weight after simvastatin treatment decreased by 44% and 31%, respectively, demonstrating that trogocytosis inhibition significantly prevented tumor progression (Figure 4O,P).

In summary, these in vivo results demonstrate that trogocytosis efficiency varies across tumor types and governs the immunosuppressive properties of the TME. Simvastatin‐mediated inhibition of trogocytosis impedes M2‐like macrophage polarization, alleviates CD8^+^ T cell exhaustion, and promotes antitumor T cell activity, supporting its potential as a strategy to improve cancer immunotherapy.

### 2.5 Trogocytosis Disruption Potentiates Adoptive Cell Transfer Therapy

Adoptive T cell therapy (ACT) is one of the main immunotherapeutic strategies in clinical [48]. However, its performance in solid tumors remains very poor, and the immunosuppressive TME is an important reason for this deficiency [49, 50]. Since trogocytosis of tumor cells by M2‐like macrophages contributes to the immunosuppression of the TME. Trogocytosis inhibition would enhance the ACT efficacy in solid tumors. To test this, CFSE‐labeled and activated OT‐I T cells were intravenously administrated into 4T1‐OVA (Ovalbumin) or MC38‐OVA tumor‐bearing mice with or without simvastatin treatment (Figure 5A).

**FIGURE 5:**
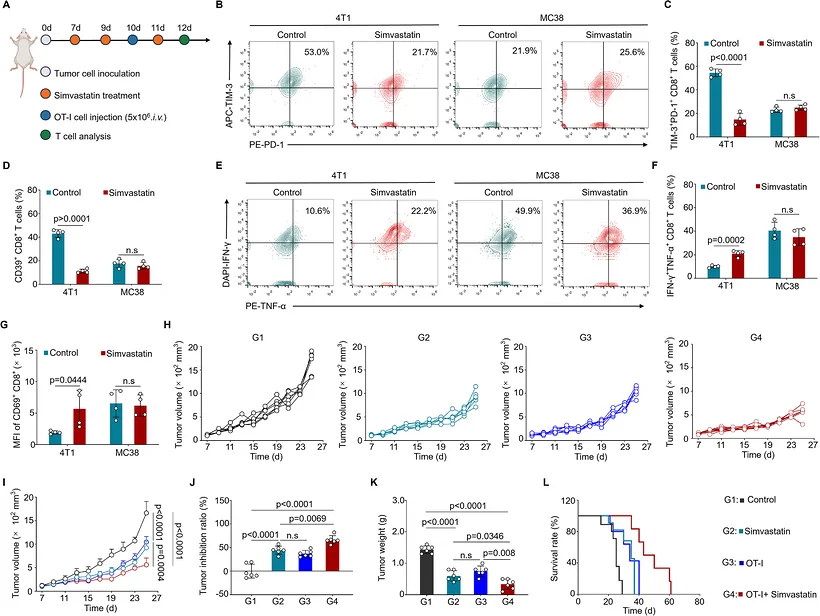
Trogocytosis disruption potentiates adoptive cell transfer therapy. (A) Schematic of 4T1‐OVA and MC38‐OVA tumor inoculation and therapeutic regimens. Tumor‐bearing mice were treated with three intraperitoneal injections of simvastatin and one intravenous injection of 5.0 × 10^6^ OT‐I T cells. (B,C,D) Quantification of TIM‐3^+^PD‐1^+^CD8^+^ T cells (B, C) and CD39^+^CD8^+^ T cells (D) in breast cancer versus colorectal cancer tissues (*n* = 4). (E,F) Representative flow cytometry and corresponding quantification of IFN‐γ^+^TNF‐α^+^CD8^+^ T cells in tumors with or without simvastatin treatment (*n* = 4). (G) Quantification of CD69^+^CD8^+^ T cells (mean fluorescence intensity, MFI) in tumors with or without simvastatin treatment (*n* = 4). (H,I) 4T1 tumor growth curves across treatment groups (*n* = 6). (J) Tumor growth inhibition rates for indicated treatments (*n* = 6). (K,L) Tumor weights (K) and survival curves of breast cancer‐bearing mice after different treatments (L) (*n* = 6). Data are presented as mean ± SD from biological replicates. Statistical significance was determined by one‐way ANOVA followed by Tukey's multiple comparisons test. n.s, no significance. Unpaired two‐tailed Student's t test for (C, F).

Flow cytometry revealed that the percentage of PD‐1^+^TIM‐3^+^CD8^+^ and CD39^+^CD8^+^ exogenous T cells reached 54.5% and 43.1% in 4T1‐OVA tumors, significantly higher than those in MC38‐OVA tumors. Simvastatin treatment reduced these populations to 21.3% and 11.3% but exhibited negligible effects in MC38‐OVA tumors (Figure 5B,C,D). In contrast, the percentage of IFN‐γ^+^ TNF‐α^+^CD8^+^ exogenous T cells in MC38‐OVA tumors was 4.1‐fold higher than in 4T1‐OVA tumors, and simvastatin treatment decreased this difference to 1.7‐fold (Figure 5E,F). A similar trend was observed in CD69^+^CD8^+^ exogenous T cells (Figure 5G). These results indicate that inhibiting trogocytosis in TME promotes the immunity of not only endogenous but also exogenous tumor infiltrating CD8^+^ T cells. Inspired by these findings, we next assessed the anti‐tumor effects of ACT in combination of trogocytosis disruption by employing a 4T1‐OVA subcutaneous tumor model. Simvastation and OT‐I T cells alone demonstrated tumor inhibition rates of 44.4% and 37%, respectively, and the combination treatment achieved an inhibition rate of 66.2% (Figure 5H–J). Tumor weight measurement and histological examination further supported these findings (Figure 5K and Figure S5A). Consequently, combination treatment significantly prolonged the survival rate of mice (Figure 5L), while it did not result in obvious adverse effects (Figure S5B,C). Therefore, trogocytosis inhibition represents a novel strategy in potentiating ACT therapy efficacy in solid tumors.

### 2.6 M2‐Mediated Trogocytosis in Human Tumors

Next, we continue to determine whether this trogocytosis and its heterogeneity observed in mice extends to human. The breast cancer cells of human (MDA‐MB‐231) were biotinylated and co‐incubated with M2‐phenotype THP‐1 macrophages at a 1:1 ratio. Flow cytometry revealed substantial membrane transfer, with 78.9% of M2 THP‐1 macrophages becoming biotin‐positive after 6 h (Figure 6A,B). In contrast, minimal biotin signal was detected in M2 THP‐1 macrophages when they were physically separated from MDA‐MB‐231 cells using a Transwell system. Furthermore, treatment with latrunculin A reduced the biotin acquisition by 3.7‐fold. These results demonstrated robust trogocytosis between MDA‐MB‐231 cells and M2 THP‐1 macrophages. We next examined trogocytosis between M2 THP‐1 macrophages and other human tumor cells, including A875 melanoma cells and LS513 colorectal carcinoma cells. Marked heterogeneity was observed: the percentage of biotin‐positive M2 THP‐1 macrophages was 31.6% for A875 and 16.5% for LS513, compared to 78.9% for MDA‐MB‐231 (Figure 6A,B). CLSM imaging confirmed a progressive decrease in biotin intensity in the order of MDA‐MB‐231, A875, and LS513 groups (Figure 6C and Figure S6A,B,C). Quantitatively, biotin signal in the MDA‐MB‐231 group was 4.0‐ and 16.5‐fold higher than that in the A875 and LS513 groups, respectively (Figure 6D). Given the established role of cortical stiffness in trogocytosis, we measured the mechanical properties of the three human tumor lines. LS513 cells displayed significantly greater cortical stiffness than MDA‐MB‐231 and A875 cells, and a strong negative correlation was observed between tumor cell stiffness and trogocytosis efficiency (Figure S6D,E; R^2^ = 0.84, r = ‐0.81, p = 0.0005). Cortical stiffness of all three tumor cells was significantly strengthen with simvastatin treatment (Figure S6D). We also assessed the expression of *CXCL5* and *Arg‐1*, both of which correlated positively with trogocytosis efficiency (R^2^ = 0.98, r = 0.99, p<0.0001 and R^2^ = 0.91, r = 0.96, p<0.0001 respectively; Figure S6F,G). qRT‐PCR analysis showed that simvastatin attenuated the upregulation of both *CXCL5* and *Arg‐1*. Specifically, in MDA‐MB‐231 cells, *CXCL5* expression increased 30.5‐fold and was reduced to 9.3‐fold with simvastatin treatment. In A875 cells, the 8.3‐fold increase was suppressed by 68.7%, and in LS513 cells, the 2.75‐fold upregulation was diminished by 42.8%. *Arg‐1* expression followed a similar pattern (Figure 6E,F). Moreover, *Arg‐1* levels were positively correlated with *CXCL5* expressions (Figure 6G, R^2^ = 0.88, r = 0.94, p = 0.0002).

**FIGURE 6:**
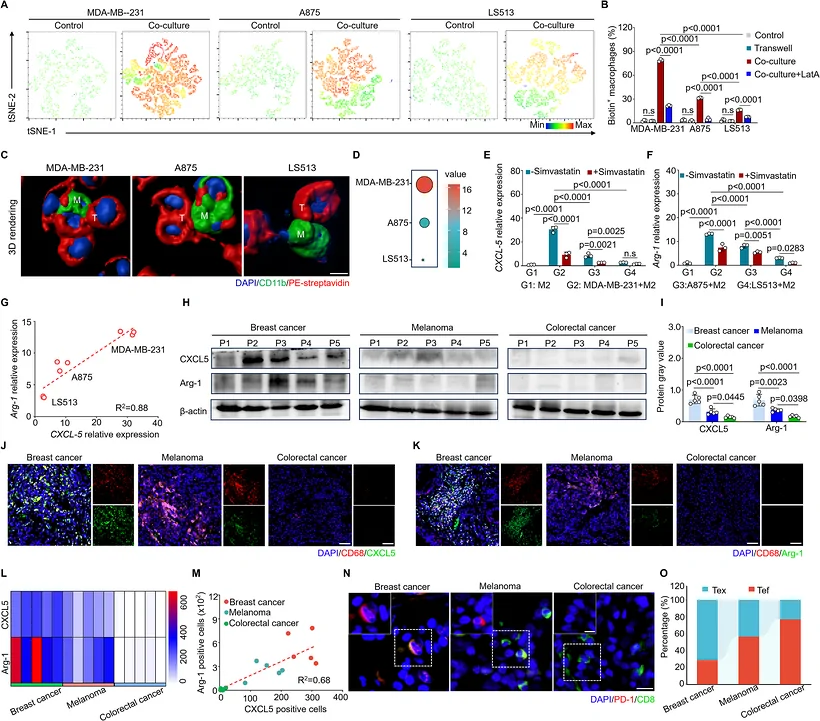
M2‐mediated trogocytosis in human tumors. (A,B) Representative flow cytometry and corresponding quantification of biotin‐positive M2 THP‐1 macrophages after 6 h of co‐culture. Cells were stained with PE‐streptavidin to detect biotinylated proteins acquired from tumor cells (*n* = 3). (C) Representative three‐dimensional reconstitution images of trogocytosis between M2 THP‐1 macrophages and three kinds of tumor cells. Nucleus (Blue), M2 macrophages (Green), tumor cells (Red). Scale bar: 10 µm (M: M2 THP‐1 macrophage; T: tumor cell). (D) Fold‐change quantification of M2 THP‐1 macrophage‐tumor cell co‐localization in (C) (*n* = 4). (E,F) Representative expression of *CXCL5* (E) and *Arg‐1* (F) in M2 THP‐1 macrophages after co‐culture with untreated or treated tumor cells for 24 h. (*n* = 3). (G) Correlation between *CXCL5* and *Arg‐1* cells across tumor lines (*n* = 3). (H,I) Western blotting analysis of CXCL5 and Arg‐1 in three tumor species (H) and quantified protein expression (I). (*n* = 5). (J,K,L) Representative immunofluorescence images and corresponding quantitative analysis of CXCL5 (J,L) and Arg‐1 (K,L) in CD68^+^ macrophages in different cancer specimens (*n* = 5). Nucleus (Blue), CXCL5 or Arg‐1 (Green), and CD68^+^ macrophages (Red). Scale bar: 50 or 100 µm. (M) Correlation between CXCL5 and Arg‐1 in different cancer specimens. (N,O) Representative fluorescence imaging and quantitative analysis of PD‐1^+^CD8^+^ T cells in different tumor types (Nucleus, blue; PD‐1, green; CD8^+^ T cell, red). Exhaustion T cells were stained with PE‐conjugated anti‐PD‐1 antibody and FITC‐conjugated anti‐CD8 antibody. Tex: exhaustion T cell; Tef: effector T cell. Scale bar: 50 µm (original images) or 25 µm (enlarged images). Data are presented as mean ± SD from biological replicates. Statistical significance was determined by one‐way ANOVA followed by Tukey's multiple comparisons test. n.s, no significance.

To further validate these findings in a clinical context, we analyzed surgically excised specimens from patients with breast cancer, melanoma, or colorectal cancer. Both western blotting and immunofluorescence confirmed a progressive decline in CXCL5 and Arg‐1 expressions in the order of breast cancer, melanoma, and colorectal cancer (Figure 6H–L). Expression levels of both proteins were significantly associated with cancer types (Figure 6M). Importantly, this gradient mirrored the extent of CD8^+^ T cells exhaustion, which was highest in breast cancer (72%), intermediate in melanoma (48%), and lowest in colorectal cancer (20%) (Figure 6N,O). Collectively, our results reveal that M2‐mediated trogocytosis also occurs in human tumors, and that trogocytosis heterogeneity across tumor types drives distinct immunosuppressive of TME, ultimately shaping the extent of CD8^+^ T cell exhaustion.

## 3 Disscusion

Immunotherapy has revolutionized cancer treatment, but its efficacy in solid tumors remains limited, largely due to the immunosuppressive TME restricts effector T cell immunity [49, 51, 52]. In this study, we identify macrophage trogocytosis of non‐opsonized tumor cells as a novel and mechanically regulated process that contributes significantly to the heterogeneous immunosuppressive landscape of the TME. The efficiency of this cell‐cell interaction varies substantially across tumor types and is governed primarily by the cortical stiffness of the tumor cells, with softer cells being more susceptible. This physical nibbling event triggers profound transcriptomic reprogramming in TAMs, leading to an efficiency‐dependent upregulation of key immunosuppressive mediators, including CXCL5 and Arg‐1. The robustness of this axis is confirmed by its conservation in human cancers and its correlation with CD8^+^ T cell exhaustion in clinical specimens.

The strong correlation between trogocytosis efficiency and T cell dysfunction suggests it is a key determinant of immunotherapy response heterogeneity. Using simvastatin to increase tumor cell stiffness effectively suppressed trogocytosis and its downstream immunosuppressive cascade. This intervention not only reinvigorated endogenous T cell immunity but also potently enhanced the efficacy of adoptive T cell therapy in a resistant tumor model. Therefore, targeting trogocytosis, either directly or through modulators of cellular mechanics, represents a promising strategy to remodel the TME and overcome immunotherapy resistance in solid tumors.

Although we establish macrophage trogocytosis as a fundamental driver of immunosuppressive heterogeneity and highlight its therapeutic potential for enhancing cancer immunotherapy, several limitations should be acknowledged. First, the depth of clinical validation remains limited. Patient tissue analysis was performed using a relatively small cohort, and the associations between trogocytosis‐related markers, CXCL5/Arg‐1 expression, and T cell exhaustion remain correlative. Future studies integrating larger patient cohorts and independent validation using public single‐cell transcriptomic datasets will be important to further establish the clinical relevance of this axis across different cancer types. Second, although our experimental approaches support the occurrence of trogocytosis in vivo, direct real‐time visualization of this process within intact tumors remains technically challenging and will require the development of advanced imaging strategies. Third, while simvastatin effectively suppressed tumor cell trogocytosis and improved antitumor immunity, its potential pleiotropic effects on other host cell populations cannot be completely excluded. Finally, the therapeutic studies were conducted over a relatively short observation period (19 days), and longer‐term studies are warranted to evaluate the durability and translational potential of this intervention. Despite these limitations, our findings establish a mechano‐immunological axis linking tumor cell mechanics to macrophage trogocytosis and immunosuppressive remodeling of the tumor microenvironment, providing a potential strategy for improving the efficacy of cancer immunotherapy.

## 4 Material and Method

### 4.1 Animal

All mice were purchased from Peking University Health Science Center (PKUHSC) Department of Laboratory Animal Science (Beijing, China). All mice were housed at a temperature of 20°C–25°C, with humidity of 50%–60% and under a 12 h light/12 h dark cycle. Feed and water were available. All mice received human care according to the animal care regulations of the Beijing Animal Ethics Association and the Ethics Committee of Beijing Institute of Technology (Approval ID: 2019‐0010‐M‐2020019).

### 4.2 Cell Lines

Tumor cell lines, 4T1(murine breast cancer), B16F10 (murine melanoma), MC38 (murine colorectal cancer), MDA‐MB‐231 (human breast cancer), A875 (human melanoma), LS513(human colorectal cancer) were cultured in DMEM supplemented with 10% FBS and 1% penicillin‐streptomycin (DMEM complete medium). THP‐1 (human macrophage) and Raw264.1 (murine macrophage) were cultured in RIMP1640 supplemented with 10% FBS and 1% penicillin‐streptomycin.

#### 4.2.1 Primary Cell

Mouse peritoneal macrophages were generated as described. C57BL/6 mice (6 weeks) were intraperitoneally injected with 1 mL of sterilized starch beef broth medium. Cells were collected by centrifugation at 800×g for 5 min and treated with red blood cell lysis buffer (BD Pharm Lyse) at 37°C for 10 min. After washing twice with PBS, the cells were resuspended in complete culture medium, seeded into a 24‐well plate, and cultured at 37°C for 24 h. IL‐4 (20 ng/ml) and IL‐13 (20 ng/ml) were added for stimulation for 48 h to induce M2 polarization. Spleen CD8^+^T cells were purified followed the manufacturer. C57BL/6 mice (8 weeks) were sacrificed and removed the spleen. A single‐cell suspension was obtained and purified by CD8^+^ T cell negative sorting kit (STEMCELL EasySep). Purified CD8^+^ T cells were seeded at a density of 10^6^ per well in 24‐well plates, and activation was stimulated for 72 h by adding soluble anti‐CD3 antibody (0.5 µg/mL) and anti‐CD28 antibody (0.5 µg/mL) to complete medium containing IL‐2 (20 ng/mL). Tumor associated macrophages (TAMs): mouse models bearing breast cancer, melanoma, or colon carcinoma were established by subcutaneous inoculation of 1 × 10^6^ respective tumor cells (4T1, B16F10, or MC38) into the right flank. BALB/c mice (6 weeks) were used for breast cancer models, while C57BL/6 mice (6 weeks) were used for the melanoma model and the colon carcinoma. When the volume of tumors reached 500 mm^3^, mice were sacrificed and tumor form the single‐cell suspension. Intratumoral lymphocytes are extracted using kits and stained with F4/80/MHC‐II. The population of F4/80^+^MHC‐II^−^ were purified by fluorescence‐activated cell sorting (FACS).

### 4.3 Biotinylation of Cancer Cell Lines Using NHS‐PEG_4_‐Biotin

The tumor cell lines were subjected to cell surface biotinylation by incubation with NHS‐PEG_4_‐Biotin (0.5 mg/mL) for 45 min at 37°C. After the reaction, unbound biotin esters were removed via three washes with DMEM. To evaluate the labeling efficiency, flow cytometry provided a quantitative measure of the biotin‐positive cell population, while confocal laser scanning microscopy (CLSM) directly visualized the distribution of biotin tags on the cell membrane.

### 4.4 In Vitro Trogocytosis Assays

1. Confocal microscopy detection: Biotinylated tumor cells and M2 macrophages were co‐incubated at a ratio of 1:1 (1×10^5^ cells) for 6 h at 5% CO_2_. After the end of the incubation, gently wash three times with PBS. Subsequently, APC‐anti‐CD11b (1:100) and PE‐streptavidin (1:100) were incubated on ice in the dark for 45 min. After three washes in PBS, add 2 µg/ml Hoechst 33342 to stain the nucleus for 15 min. Finally, multi‐channel scanning was performed using a laser confocal microscope to analyze the spatial distribution of tumor‐derived biotin signals within M2 macrophages by Z‐stack imaging (layer thickness 0.5 µm).
2. Flow cytometry quantification: The co‐culture system was performed in a 24‐well plate. After 6 h, the mixture cells were collected by centrifuge at 500 ×g for 5 min. After the cells were stained by APC‐anti‐CD11b and PE‐streptavidin on ice for 45 min. Resuspend the cells with 300 µL PBS after three washes. The proportion of CD11b^+^/Streptavidin^+^ double‐positive cell population was analyzed using BD Flow cytometry to further quantify the trogocytosis efficiency.
3. Flow cytometry imaging: three types of tumor cells were stained with CFSE and seeded in 12‐well plates at a density of 5 × 10^5^ cells per well. Subsequently, these cells were co‐cultured with M2 macrophages for 6 h. For confirmation of single‐cell acquisition of biotin using imaging cytometry, these co‐cultured cells were collected and stained with APC‐anti‐CD11b and PE‐ streptavidin to detect the biotin acquired directly from various tumor cells. The single‐cell images were analyzed by IDEAS software, and the fluorescence intensity and number of PE‐streptavidin on the surface of macrophages were calculated to confirm the differences in the transfer of membrane components.
4. Live‐cell imaging system: biotinylated tumor cells were stained with PE‐streptavidin, M2 macrophages were labeled with APC‐anti‐CD11b, and incubated at 37°C for 30 min in the dark, respectively. After three PBS washes, the two cells were mixed and seeded in a 1:1 ratio on 24‐well glass‐bottom plates (Cellvis) with a final density of 2×10^4^ cells/well. Immediately placed in the YOKOGAMA CV8000 (37°C, 5%CO_2_), Z‐stack images were acquired every 30 min to track the dynamic transfer of cell membranes. M2‐like Raw264.7 macrophages was considered trogocytosis‐positive when biotin transferred from tumor cells was detected in the macrophage during live‐cell imaging.

### 4.5 Analysis of M2 Macrophages Proteomic

Following 6‐h co‐culture of three distinct biotinylated tumor cell lines with M2 macrophages at a 1:1 ratio, a multi‐step procedure was employed to isolate and identify the proteins acquired by M2 macrophages via trogocytosis. Cell sorting and lysis: after the co‐culture period, M2 macrophages were specifically sorted using FACS based on established surface markers (CD11b^+^). The sorted macrophages population was lysed using a RIPA buffer supplemented with protease and phosphatase inhibitors to extract total protein. Affinity purification of biotinylated proteins: the biotinylated proteins, which originated from the tumor cell membranes and were transferred to the M2 macrophages, were isolated from the total protein lysate using streptavidin‐conjugated magnetic beads. Briefly, the protein lysate was incubated with the beads for overnight at 4°C with gentle rotation. The bead‐bound complexes were subsequently washed thoroughly to eliminate non‐specifically bound proteins. Protein elution and proteomic preparation: the specifically captured biotinylated proteins were eluted from the beads by glycine buffer (0.1 m pH 2.0). Then the enrichment proteins were analyzed by liquid chromatography coupled with tandem mass spectrometry (LC‐MS/MS).

### 4.6 Measurement of Membrane Stiffness

#### 4.6.1 Microspheres Modification

Surface modification of 1.9 µm carboxylated polystyrene microspheres to enhance their adhesion to cell membranes. EDC (final concentration 5 mm) and NHS (final concentration 10 mm) were added to the microsphere solution and reacted for 15–30 min at room temperature to activate the carboxyl group. A final concentration of 20 mm glycine was then added and incubated for 15 min at room temperature to quench the unreacted activating groups. After several centrifugation washes, a surface‐activated microsphere suspension was obtained.

#### 4.6.2 Optical Tweezers Measurement

The target tumor cells are added dropwise into a special optical glass‐bottom Petri dish, and after the cells are adherent and stable, the functionalized microsphere suspension is added at a ratio of 1:1000 to ensure that the density of the microspheres is moderate. Under an inverted microscope equipped with a light tweezers module, manipulate the light tweezers to capture individual spheroids and make them contact with the target cell membrane to form a stable connection.

### 4.7 F‐Actin Staining

The three kinds of tumor cells were seeded in glass at a density of 2 × 10^5^ cells per well. After 24 h, the tumor cells were fixed with 4% paraformaldehyde and stained with PE‐Phalloidin (1:500). The F‐actin of fluorescence intensity were measured by ImageJ.

### 4.8 Measurement of Sialic Acid

Three types of tumor cells were first subjected to enzymatic desialylation. Briefly, the tumor cells were harvested and washed with PBS. The cell pellets were then resuspended in a treatment solution containing 0.1 U/mL neuraminidase (sialidase) and incubated for 1 h at 37°C in CO_2_ incubator. Following incubation, the cells were washed thoroughly three times with complete medium to terminate the reaction and remove any residual enzyme.

The desialylated tumor cells were co‐cultured with M2 macrophages at a 1:1 ratio for 6 h. After co‐culture, the cells were analyzed by flow cytometry. The occurrence of trogocytosis was confirmed by detecting a double‐positive macrophage population, which signified the acquisition of the biotin signal from the tumor cells.

### 4.9 RNA‐Seq Analyses

Cell Sorting: CD11b^+^ macrophages populations were purified by FACS after co‐culture with various tumor cell lines. RNA preparation and sequencing: total mRNA was extracted from sorted cells using TRIzol reagent, ensuring high integrity. Sequencing libraries were constructed and profiled by high‐throughput RNA sequencing (RNA‐seq). Bioinformatics analysis: we identified differentially expressed genes (DEGs) and conducted functional enrichment analyses, including gene ontology (GO) and Kyoto Encyclopedia of Genes and Genomes (KEGG), to pinpoint key signaling pathways and biological processes altered in macrophages.

### 4.10 Measurement of Macrophages Migration

Cell migration was assessed using 24‐well Transwell chambers with polycarbonate membranes (8.0 µm pore size). Briefly, 2 × 10^4^ macrophages cells in 200 µL of suspension were seeded into the upper chamber. The lower chamber was filled with 600 µL of supernatant, which was collected from three distinct co‐culture systems and served as the chemoattractant. After incubating at 37°C for 48 h, the non‐migrated cells on the upper surface of the membrane were carefully removed with a cotton swab. The cells that had migrated to the lower surface were fixed with 4% paraformaldehyde for 15 min, stained with 0.1% crystal violet for 20 min, and then rinsed with PBS. The stained cells were visualized and photographed under an inverted microscope. Finally, the number of migrated cells was quantified by counting in three random fields per well.

### 4.11 In Vitro Trogocytosis Assays with Human Cell Lines

For confirming the trogocytosis with human cell lines, human cancer cell lines co‐culture with M2 THP‐1 macrophages as “in vitro trogocytosis assays”.

### 4.12 Immunoblotting Analysis

The human cancer specimens were crushed and obtained the protein. Protein samples were separated with SDS polyacrylamide gel electrophoresis and blotted onto nitrocellulose (NC) membrane (Millipore). The membrane was blocked with 5% BSA for 3 h and incubated with anti‐CXCL5 antibody (1:1000), anti‐Arg‐1 (1:2000), or anti‐β‐Actin antibody (1:5000) overnight at 4°C. After washing with phosphate‐buffered saline with Tween20 (PBST) for three times, the membrane was further incubated with a diluted secondary antibody Goat‐Anti‐Rabbit IgG (HRP) (1:10 000) for 2 h. Then the membrane was washed by PBST three times and visualized with ECL reagent (Thermo Scientific) by chemiluminescence system (ChemiDoc XRS+ System, Bio‐Rad).

### 4.13 Detection of CD8^+^ T Cells Exhaustion

Activated CD8^+^ T cells were incubated with sorted M2 macrophages in a 1:1 ratio for 24 h. Pooled cells after co‐culture were collected and stained for 45 min at 4°C in the dark using FITC‐anti‐CD8 antibody (1:100), PE‐anti‐PD‐1 antibody (1:100), and APC‐anti‐CD39 antibody (1:100). After staining, wash three times with pre‐chilled PBS and resuspend. Flow cytometry was used to analyze the expression levels of exhaustion marker.

### 4.14 In Vivo Immune Activation Assay

The melanoma(B16F10)‐, colon carcinoma (MC38)‐ or breast cancer(4T1)‐bearing mouse model was established. When the volume of tumors reached 100 mm^3^, simvastatin was Intraperitoneal administration into mice every two days for three consecutive administrations. After completing three rounds of dosing, the rats were sacrificed. For immune cell analysis, tumors were cut into small pieces and then homogenized in cold PBS to form the single‐cell suspension. The obtained cells were divided into several parts to analyze different immune cell types, respectively. For total T cells infiltration analysis, cells from tumors were stained with CD3, CD8, CD4 antibodies. For cytotoxic T lymphocytes (CTL) analysis, cells from tumors were stained with CD3, CD8, IFN‐γ TNF‐α antibodies. For T cells exhaustion analysis, cells from various tumors were stained with CD3, CD8, PD‐1, TIM‐3, and CD39. Then, stained cells were washed with PBS three times and measured by flow cytometer (Bioscience FACSAria, BD).

### 4.15 Co‐Transplantation of M2 Macrophages and Cancer Cells

For detection trogocytosis in vivo, the M2 macrophages co‐culture with 4T1 cells. After 24 h, the sample were collected and subcutaneously injected into the right flank in BALB/c mice. After 7 days, the tumor volumes and body weights were measurement every two day. The tumor volumes were calculated by the formula of (L × W^2^)/2.

### 4.16 In Vivo Antitumor Activity

For 4T1 tumor inhibition, 1 × 10^6^ 4T1‐OVA was subcutaneously injected into the right flank in BALB/c mice. When the tumor volume reached about 50 mm^3^, mice were randomly divided into four groups, and treatment with PBS, Simvastatin, OT‐I, Simvastatin+OT‐I (Simvastatin dose: 25 mg/kg; OT‐I: 5 × 10^6^ per mouse). The length (L) and width (W) of the subcutaneous tumors and the body weights were measured every other day after the first administration. The tumor volumes were calculated by the formula of (L × W^2^)/2. When the tumor volume was larger than 1500 mm^3^, the mice were euthanized by cervical dislocation. For safety evaluation, at the end of therapy, some mice were sacrificed, and the main organs (heart, liver, spleen, lung, and kidneys) were harvested, fixed in 4% paraformaldehyde solution, and sectioned into slices for the hematoxylin and eosin (H&E).

### 4.17 Clinical Study

The surgically excised specimens from melanoma, colon carcinoma, and breast cancer patients were collected (*n* = 7). Three kinds of clinical tissue proteins were extracted with a protein extraction kit. Following this, the expression levels of CXCL5 and Arg‐1 were analyzed by western blotting (Primary antibody: human anti‐CXCL5 monoclonal antibody (1:1000, 4°C, overnight), human anti‐Arg‐1 monoclonal antibody (1:2000, 4°C, overnight), or human anti‐β Actin monoclonal antibody (1:10 000, 4°C, overnight); secondary antibody: HRP‐conjugated Goat Anti‐Mouse IgG(H+L) (1:10 000, RT, 120 min) or HRP‐conjugated Goat Anti‐Rabbit IgG(H+L) (1:10000, RT, 120 min)). For immunofluorescence, the paraffin sections of human cancer tissues were stained with anti‐human CXCL5 antibody (1:500, 4°C, overnight), FITC‐anti‐human Arg‐1 antibody (1:500, 4°C, overnight), FITC‐anti‐human CD8a antibody (1:500, 4°C, overnight), PE‐anti‐human PD‐1 antibody (1:500, 4°C, overnight). Secondary antibody: FITC‐conjugated Goat Anti‐Mouse IgG(H+L) (1:1000, RT, 2 h).

### 4.18 Statistical Analysis

An unpaired two‐tailed student t‐test was used to analyze the statistically significant differences and data were considered statistically significant when the values of *p* < 0.05. ^*^ *p* < 0.05, ^**^ *p* < 0.01, ^***^ *p* < 0.001, and ^****^ *p* < 0.0001. n.s., no significant. Comparisons among three or more groups were performed using one‐way analysis of variance (ANOVA). Regression coefficients were assessed using Wald tests, and 95% confidence intervals are reported. Image Lab was used to analyze the data of western blot. FlowJo (version number, 10.0.0.0) was used to analyze the data of flow cytometry. GraphPad Prism (version number, 9.0.2.263) was used for the statistical analysis.

## Author Contributions

Conceptualization: H.X., C.L., and Y.L. Methodology: C.L., J.H., and W.Z., Z.J. Investigation: C.L., Y.L., Y.J., and Y.L. Visualization: C.L., X.M., and P.L. Funding acquisition: H.X., W.Z., Y.L. Project administration: H.X. Supervision: H.X., C.L., and Y.L. Writing – original draft: H.X., C.L., and Y.L. Writing – review & editing: H.X., C.L., and Y.L.

## Conflicts of Interest

The authors declare no conflicts of interest.

## Supporting information




**Supporting File**: advs77518‐sup‐0001‐SuppMat.docx.

## Data Availability

The data that support the findings of this study are available from the corresponding author upon reasonable request.
