# Supplementary material for: Macrophage Trogocytosis of Tumor Cells Drives the Immunosuppression of Tumor Microenvironment
Source: Adv Sci (Weinh). 2026 Sep 30:e77518. Online ahead of print. doi: 10.1002/advs.77518 (PMC13625920; doi:10.1002/advs.77518)
Supplement: Supplementary file 1 [file ADVS-9999-e77518-s001.docx]

**Macrophage trogocytosis of tumor cells drives the immunosuppression of tumor microenvironment**

Chao Liang^1,3^, Jiaqi He^4^, Wan-Ru Zhuang^1^, Yao Lei^1^, Yanru Jiang^1^, Xianbin Ma^5^, Pan Liu^1^, Zhujun Jiang^1^, Yongheng Li^2^*, Hai-Yan Xie^1^*

^1^State Key Laboratory of Natural and Biomimetic Drugs, School of Pharmaceutical Sciences, Chemical Biology Center, Peking University, Beijing 100191, China

^2^Key laboratory of Carcinogenesis and Translational Research (Ministry of Education/Beijing), Department of Radiation Oncology, Peking University Cancer Hospital and Institute, Beijing 100142, China

^3^Department of Pancreatic Cancer, Tianjin Medical University Cancer Institute and Hospital, National Clinical Research Center for Cancer, Tianjin Key Laboratory of Digestive Cancer, Key Laboratory of Cancer Prevention and Therapy, 300060, Tianjin, China

^4^Division of Life Science and Medicine, University of Science and Technology of China, Hefei 230026, China

^5^School of Medical Technology, Beijing Institute of Technology, Beijing 100081, China

**Supplementary Figure 1. Tumor-type-specific hierarchy of trogocytosis by macrophages. A.** Representative CLSM of phagocytosis between M2 macrophages and three kinds of tumor cell lines. Scale bar:100 μm. **B.** Representative biotin distribution in tumor cells stained with PE-streptavidin after NHS-PEG_4_-Biotin treatment. Scale bar: 40 μm. **C.** Representative CLSM of trogocytosis between M2 macrophages and three kinds of tumor cells. Nucleus (Blue), M2 macrophages (Green), tumor cells (Red). Scale bar: 20 μm. **D.** Quantitative the total number of cells before and after co-culture (n=3). **E.** A schematic diagram of the experimental design for investigating trogocytosis between tumor-associated macrophages and tumor cells. **F.** Representative CLSM of trogocytosis between tumor-associated macrophages and three kinds of tumor cells. Scale bar: 10 μm.

**Supplementary Figure 2. Membrane stiffness hierarchy determines trogocytosis intensity. A.** Representative flow cytometry and corresponding quantification of Dsg-1 in M2 macrophages after 6h co-culture (n=3). **B.** Representative flow cytometry and corresponding mean fluorescence intensities (MFI) of Dsg-1 in different tumor cells (n=3).  **C.** Dsg-1 expression in M2 macrophages after co-culture with three kinds of tumor cells (n=4). **D.** Representative flow cytometry and corresponding mean fluorescence intensities (MFI) of sialic acid after sialidase treatment (n=3). NA：Neuraminidase. **E.** Representative flow cytometry and corresponding quantification of biotin+ M2 macrophages co-culture with tumor cell treated with NA (n=3). **F.** Correlation between membrane stiffness and trogocytosis efficiency with three kinds of tumor cells after simvastatin treatment (n=3).

**Supplementary Figure 3. Trogocytosis-driven macrophage polarization and recruitment promotes T cell exhaustion. A.** Relative mRNA levels of cytokines (**left**) and metabolic regulators (**right**) in M2 macrophages separated with various tumor cell by Transwell system. **B.** Correlation between trogocytosis efficiency and the expression of CXCL5(left) and Arg-1(right). **C.** Representative flow cytometry and corresponding mean fluorescence intensities (MFI) of Arg-1 in M2 macrophages after co-culture with untreated or treated tumor cells for 24 h. **D.** M0 Raw264.7 macrophage recruitment quantification under different supernatant treatments. E. Correlation between migrating cells and the expression of CXCL5. **E.** Correlation between trogocytosis efficiency and migrating cells (n=3). **F.** Correlation between PD-1^+^CD8^+^ T cells trogocytosis efficiency with three kinds of tumor cells (n=3). **G.** Correlation between CD39^+^CD8^+^ T cells and trogocytosis efficiency with three kinds of tumor cells (n=3).

**Supplementary Figure 4.** **Simvastatin disrupts trogocytosis and reverses T-cell exhaustion *in vivo.* A.** Flow cytometric analysis of Dsg1^+^ TAMs in tumors with or without simvastatin treatment (n = 4). **B**. Correlation between Dsg1^+^F4/80^+^ cells and *CXCL5* expression across tumor lines. **C.** Correlation between Dsg1^+^F4/80^+^ cells and *Arg-1* expression across tumor lines. **D.** Correlation between F4/80^+^CD206^+^ cells and *CXCL5* expression across tumor lines. **E.** Correlation between Dsg1^+^F4/80^+^ cells and CD3^+^T cells across tumor lines. **F.** Flow cytometric analysis of IFN-γ^+^ TNF-α^+^CD8^+^ T cells in tumors with or without simvastatin treatment (n = 4). **G.** Scheme: Trogocytosis inhibition reprograms the tumor immune microenvironment.

**Supplementary Figure 5. Trogocytosis disruption potentiates adoptive cell transfer therapy. A.** H&E staining of tumor sections after different treatments. Scale bar: 100 μm**. B.** Body weight during antitumor experiment against4t1-OVA (n=6). **C.** H&E staining of major organs from mice at the endpoint of the 4T1 tumor model. Scale bar: 400 μm

**Supplementary Figure 6. M2-mediated trogocytosis in human tumors. A.** Representative CLSM of trogocytosis between M2 THP-1 macrophages and three kinds of tumor cells. Nucleus (Blue), macrophages (Green), tumor cells (Red). Scale bar: 20 μm. **B.** Representative three-dimensional images of trogocytosis between M2 THP-1 macrophages and three kinds of tumor cells. Nucleus (Blue), macrophages (Green), tumor cells (Red). Scale bar: 10 μm**. C.** Correlation between trogocytosis frequency and biotin intensity. **D.** Quantification of membrane stiffness in various tumor cell lines with or without simvastatin treatment. (n=3)**. E.** Correlation between membrane stiffness and trogocytosis efficiency (n=3)**. F.** Correlation between CXCL5 expression and trogocytosis efficiency (n=3)**. G.** Correlation between Arg-1 expression and trogocytosis efficiency (n=3).

**Supplementary Figure 7. The gating strategies for the flow cytometry analyses*.*** (n=4)**. A.** Gating strategy for flow cytometric studies of CD4^+^ T cells and CD8^+^ T cells (gated on CD3^+^ T cells) in tumors. **B.** Gating strategy for flow cytometric studies of IFN-γ^+^ TNF-α^+^T cells (gated on CD8^+^ T cells) in tumors. **C.** Gating strategy for flow cytometric studies of exhaustion markers (gated on CD8^+^ T cells) in tumors.
